# Verteporfin Inhibits Severe Fever with Thrombocytopenia Syndrome Virus Infection via Inducing the Degradation of the Viral Gn Protein

**DOI:** 10.3390/pharmaceutics17040434

**Published:** 2025-03-28

**Authors:** Bingan Wu, Chenyang Yu, Yuxiang Lin, Ping Zhao, Zhongtian Qi, Xijing Qian

**Affiliations:** 1 Department of Microbiology, Faculty of Naval Medicine, Naval Medical University, Shanghai 200433, China; wuba@smmu.edu.cn (B.W.); 18069886238@163.com (C.Y.); pnzhao@163.com (P.Z.); 2 College of Basic Medical Sciences, Naval Medical University, Shanghai 200433, China; 13625015535@163.com

**Keywords:** severe fever with thrombocytopenia syndrome virus, antiviral agents, verteporfin, viral binding, Gn protein

## Abstract

**Background:** Severe fever with thrombocytopenia syndrome virus (SFTSV) is a novel tick-borne bunyavirus, causing the hemorrhagic infectious disease of SFTS, with a case fatality rate up to 30% due to the absence of effective therapeutic interventions. Therefore, it is urgent to develop safe and effective therapeutic drugs to control this viral hemorrhagic fever. **Methods:** The activity of verteporfin (VP), screened from an FDA-approved drugs library, against SFTSV, was systematically evaluated in Huh7 cells in a wide range of concentrations. We performed time-of-addition experiments with VP, along with binding, endocytosis, and membrane fusion assays, to determine which part of the SFTSV life cycle VP has its effect on. The potential targets of VP were detected by a drug affinity responsive target stability (DARTS) assay. **Results:** VP exhibited a potent anti-SFTSV activity by blocking the initial viral binding to the target cells during viral entry via significantly inducing the degradation of the viral Gn protein. **Conclusions:** The VP-induced inhibition of SFTSV binding, the first step of viral invasion, suggested that VP might be an ideal and potent anti-SFTSV agent due to its prophylaxis and therapeutic effects on viral infection.

## 1. Introduction

Severe fever with thrombocytopenia syndrome (SFTS), caused by the novel identified bunyavirus (SFTS virus, SFTSV) in the family Phenuiviridae of the order Bunyavirales, is an emerging tick-borne viral hemorrhagic fever disease [1]. SFTSV cases have been reported in many countries, such as China, Japan, Korea, and Vietnam [2] since its initial identification in China in 2009 [3]. It is thought that the tick bites are the primary way to transmit SFTSV [4], though human-to-human contact through direct exposure to body fluids of SFTS cases have also been a potential transmission route [5]. SFTS patients mainly present with fever, thrombocytopenia, leukocytopenia, gastrointestinal symptoms, or even multiple organs failure [6] with a high case fatality rate of 12–30% [7]. This situation is mainly due to the absence of effective drugs against SFTSV, posing a serious threat to public health worldwide [8]. In recent years, environmental changes have also caused a remarkable expansion of tick populations and their habitats [9], resulting in a potential global outbreak of SFTS. Therefore, the SFTS has been listed as a priority infectious disease by the World Health Organization (WHO) in 2024 [10], calling for effective therapeutic strategies.

SFTSV is an enveloped negative-sense RNA virus with a tripartite genome of large (L), medium (M), and small (S) segments. These genome segments encode RNA-dependent RNA polymerase (L), glycoprotein precursor (M), nucleoprotein, and nonstructural proteins (S), respectively [11]. The glycoprotein precursor will be further cleaved into the N-terminus fragment (Gn) and the C-terminus fragment (Gc), which are the two major viral antigenic components to prompt the production of specific neutralizing antibodies in the host body against SFTSV [12,13]. Furthermore, Gn and Gc play critical roles in viral entry. It has been indicated that Gn mediates the initial binding of SFTSV to the receptors of target cells, and Gc is responsible for the subsequent membrane fusion process [14,15]. More importantly, a recent study identified that Gn could facilitate the immune escape of SFTSV via the inhibition of the innate immune responses through the suppression of the cyclic GMP-AMP synthase (cGAS)-stimulator of interferon genes (STING) pathway and the induction of STING degradation [16]. Considering the immunogenicity and critical role of the Gn protein in SFTSV infection, it has been identified as a significant target for the development of anti-SFTSV drugs and vaccines.

Verteporfin (VP), a derivative of benzoporphyrin, is commonly used as a photosensitizer for the treatment of the choroidal neovascularization associated with age-related macular degeneration, ocular histoplasmosis, and pathologic myopia [17]. VP has also been extensively studied as an anti-tumor agent due to its capacity of directly binding to the yes-associated protein (YAP) and disruption of the interaction between the YAP and transcriptional enhanced associate domain (TEAD) [18]. Moreover, it has been demonstrated by several studies that VP exerted inhibitory activity against a variety of viruses, such as human cytomegalovirus, human immunodeficiency virus, and severe acute respiratory syndrome coronavirus 2 [19,20,21]. However, no study has been made on the bunyaviruses.

In the present study, we have identified VP with significant anti-SFTSV activity via high-throughput screening. Further experiments revealed that VP suppressed SFTSV infection by inhibiting viral binding during the early entry stage of the viral life cycle probably via the degradation of viral Gn. SFTSV binding to cell surface receptors is the first step to SFTSV invasion. Thus, the influence of VP on viral binding suggested its potential for utilization as a pharmaceutical anti-SFTSV agent for the prevention and treatment of SFTSV infection.

## 2. Materials and Methods

### 2.1. Cells and Viruses

The following cell lines were utilized in this study: African green monkey kidney Vero cells (ATCC CCL-81) and human hepatoma Huh7 cells (SCSP-526, Chinese Academy of Sciences, Beijing, China). Vero and Huh7 cells were cultured in Dulbecco’s modified Eagle’s medium (DMEM; Gibco, Carlsbad, CA, USA) with 10% fetal bovine serum, 1% L-glutamine, 1% nonessential amino acids, and 100 IU/mL penicillin–streptomycin (Gibco, Carlsbad, CA, USA). All cells were incubated at 37 °C in 5% CO_2_. The SFTSV was isolated from the serum of a confirmed SFTS patient in our biosafety level 3 laboratory and the whole genome sequencing revealed that the virus belongs to the SFTSV strain JS2012-70. The CHIKV LR2006 OPY1 (DQ443544) strain was synthesized in this laboratory. The Zika virus (ZIKV, strain GZ01) was stored in our laboratory. The YFV strain FJYF03/2016 was isolated from the serum of a confirmed YF patient (GenBank: KY587416.1) in our biosafety level 3 laboratory. The infectious cDNA clone of a West Nile virus (WNV) isolate WNNY 2000-crow3356 (GenBank: AF404756.1) was established in this laboratory. ZIKV was propagated in C6/36 cells and other viruses were propagated in Vero cells.

### 2.2. Chemicals and Antibodies

Verteporfin (VP, S1786) and bafilomycin (S1413) were purchased from Selleck. Cell Counting Kit-8 (CCK-8) was procured from Invitrogen (Carlsbad, CA, USA). Anti-SFTSV nucleoprotein (NP) mouse monoclonal antibody (mAb) was prepared by the Huadong Medical Institute of Biotechniques (Nanjing, Jiangsu, China). Anti-CHIKV E polyclonal antibody (pAb), anti-ZIKV NS1 pAb, anti-YFV NS1 pAb, and anti-WNV NS1 pAb was prepared in our laboratory. Ammonium Chloride (NH_4_Cl) and 3,3′-Dioctadecyloxacarbocyanine perchlorates (DiO) were obtained from the Sigma-Aldrich Corporation (St Louis, MO, USA). 4′,6-diamidino-2-phenylindole (DAPI), Alexa Fluor™ 488-Transferrin (TF) and Alexa Fluor™ 488 secondary antibody were purchased from Invitrogen (Carlsbad, CA, USA). Pronase from Merck (Darmstadt, Germany) was used in this study.

### 2.3. Immunofluorescent (IF) Assay

The SFTSV, CHIKV, ZIKV, YFV, or WNV infected cells were fixed using cold methanol at −20 °C for 30 min and then incubated with 3% bovine serum albumin at room temperature for 2 h for blocking. Next, the cells were stained with the primary anti-SFTSV NP mAb, anti-CHIKV E pAb, anti-ZIKV NS1 pAb, anti-YFV NS1 pAb, or anti-WNV NS1 pAb and an appropriate Alexa Fluor™ 488 secondary antibody. Nuclei were stained with DAPI and then detected by the BioTek Lionheart FX Imaging Reader (Agilent, Santa Clara, CA, USA).

### 2.4. Cytotoxic Assay

The VP cytotoxicity was assessed using the Cell Counting Kit-8 (CCK-8) reagent. Briefly, Huh7 cells seeded in 96-well plates were treated with indicated concentrations of VP for 24 h. Then, the supernatant was replaced with a fresh culture medium containing the CCK-8 reagent and incubated with the cells at 37 °C for 1 h [22], after which the optical density (OD) of each well was measured at 450 nm using a microplate reader. Dose–response curves were generated using Prism 9.5 (GraphPad software) to calculate the half-maximal cytotoxic concentration (CC_50_).

### 2.5. Time Window of Compound Affecting Assay

Huh7 cells were incubated with SFTSV (MOI = 1) at 37 °C and treated with VP (4 μM) for 2 h time points either 2 h prior to infection, simultaneously with the SFTSV infection (0 h), or at 2 h intervals after the virus infection up to 12 h. The amount of viral infection was determined by IF. 

### 2.6. Real-Time Quantitative Polymerase Chain Reaction (RT-qPCR)

The Huh7 cells infected with SFTSV were lysed using a TRIzol reagent (Takara, Shiga, Japan) to extract the total cellular RNA according to the instructions of the reagent. Then, the RNA was subjected to reverse transcription via a PrimeScript^TM^ RT Master Mix kit (Takara, Japan) and the cDNA was subsequently detected using a TB Green Premix ExTaq^TM^ Kit (Takara, Japan) in Applied Biosystems Quant Studio 3 (Foster City, CA, USA). The GAPDH was utilized as an endogenous control for the normalization of SFTSV RNA levels. The primer sequences are shown in Table 1 as follows:

### 2.7. SFTSV Binding Assay

Huh7 cells were incubated with SFTSV (MOI = 5) together with indicated concentrations of VP on ice for 1.5 h. Cells were then washed three times with cold PBS to remove the free viral particles. A portion of the infected cells were lysed to detect bound SFTSV by RT-qPCR, and the other portion of the infected cells were maintained in fresh medium at 37 °C for 24 h to assess the bound virions by IF assay.

### 2.8. Endocytosis Assay

Huh7 cells were co-incubated with Alexa Fluor™ 488-labelled transferrin (TF) and VP (4 μM) or PP2 (20 μM) at 37 °C for 4 h. Then, cells were fixed with 4% paraformaldehyde (PFA) at room temperature for 10 min and the nuclei were stained with DAPI. Fluorescent intensity, reflecting TF internalization, was detected using the BioTek Lionheart FX Imaging Reader (Agilent, Santa Clara, CA, USA).

Alternatively, Huh7 cells seeded in 12-well plates were incubated with SFTSV on ice for 1.5 h. Then, the supernatant was replaced with a fresh culture medium with VP and incubated at 37 °C for 1 h. The cells were then treated with 1 mg/mL proteinase K to remove the uninternalized SFTSV and washed three additional times with cold PBS. The internalized viral RNA levels were analyzed via an RT-qPCR assay.

### 2.9. Membrane Fusion Assay

The SFTSV (6.0 × 10^7^ PFU/mL) was labelled with DiO (the final concentration of DiO was 10 μM) in the dark at room temperature for 1 h. Then, Huh7 cells were infected with the labeled SFTSV (SFTSV^DiO^) in the presence of VP (4 μM), bafilomycin A1 (20 nM) or NH_4_Cl (20 mM) in two parallel 96-well plates. One plate was fixed with PFA after incubation at 37 °C for 2 h. Then, the DiO fluorescent intensity was measured using the BioTek Lionheart FX Imaging Reader. The other plate was transferred to a BioTek Synergy H1 microplate reader for a real-time monitoring the fluorescent intensity of DiO every 15 min for a total of 8 h, in which the excitation and emission were 484 nm and 501 nm, respectively.

### 2.10. Drug Affinity Responsive Target Stability Assay (DARTS)

The DARTS assay was performed according to a previous study [23]. Briefly, the Gn protein was pre-treated with VP at room temperature for 1 h. Then, the mixture was incubated with pronase at room temperature for 30 min with a ratio of pronase verses protein to be 1:100 (1 μg pronase per 100 μg protein). The influence of VP on the Gn protein was subsequently analyzed via a Western Blotting assay.

### 2.11. Statistical Analysis

The continuous variables were shown as means and standard deviations (SD) in at least three independent experiments. The Student’s *t*-test or the nonparametric Wilcoxon rank-sum test were used for comparisons between two groups. All statistical analyzes were performed using GraphPad Prism 9.5 software and a 2-sided *p* value of less than 0.05 was considered statistically significant.

## 3. Results

### 3.1. VP Has a Dose-Dependent Inhibition Effect on SFTSV Infection

**Figure 1:**
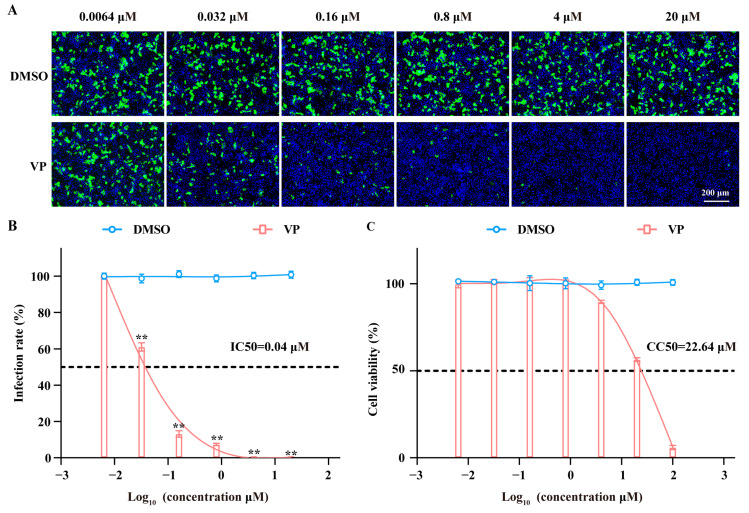
VP displays dose-dependent anti-SFTSV activity and low cytotoxicity. (**A**,**B**) Huh7 cells were incubated with SFTSV (MOI = 1) in the presence of VP or DMSO at 37 °C for 24 h. The inhibition rate of VP was analyzed via an IF assay. The SFTSV was stained with the primary anti-SFTSV NP mAb and goat anti-mouse Alexa Fluor™ 488 secondary antibody (green). Nuclei were stained with DAPI (blue). (**C**) Huh7 cells were incubated with the indicated concentrations of VP at 37 °C for 24 h, and the cytotoxicity was detected by a CCK-8 assay. Data were shown as means with a SD of three independent experiments. Scale bar, 200 μm. ** *p* < 0.01 compared to the control group.

Through high-throughput screening of the FDA-approved compound library, we have identified that verteporfin (VP) exerted a significant anti-SFTSV effect. Further investigations were conducted to ascertain the anti-SFTSV activity and cytotoxicity of VP at a broad range of concentrations. As shown in Figure 1, VP displayed a notable inhibition effect against SFTSV infection in a dose-dependent manner, of which the 50% maximal inhibitory concentration (IC_50_) and 50% cytotoxic concentration (CC_50_) were 0.04 μM and 22.64 μM, respectively, making selective index (SI) to be 566 as an ideal antiviral drug.

### 3.2. VP Suppresses SFTSV Infection via Blocking Viral Binding During the Early Entry Stage

**Figure 2:**
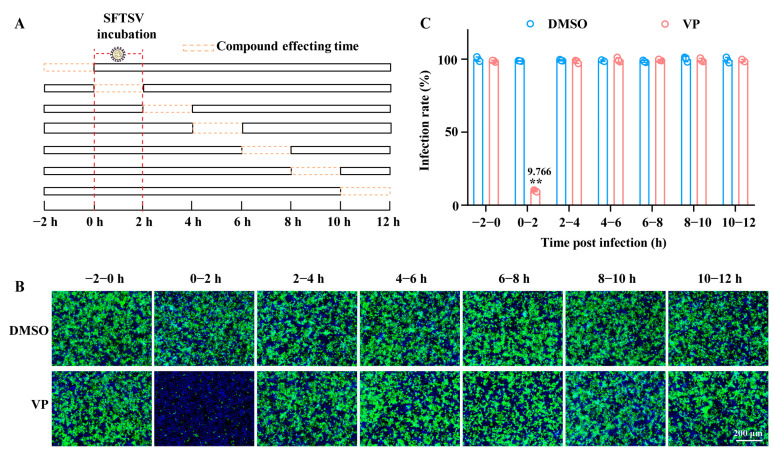
VP acts during the 0–2 h time window of the SFTSV infection. (**A**) A schematic diagram of the time window of the compound-affecting assay. (**B**,**C**) Huh7 cells were incubated with SFTSV (MOI = 1) at 37 °C for 2 h and treated with VP (4 μM) during −2–0, 0–2, 2–4, 4–6, 6–8, 8–10, and 10–12 h, respectively. The cells were detected by an IF assay 12 h after the virus inoculation. The SFTSV was stained with the primary anti-SFTSV NP mAb and goat anti-mouse Alexa Fluor™ 488 secondary antibody (green). Nuclei were stained with DAPI (blue). Data were shown as means with a SD of three independent experiments. Scale bar, 200 μm. ** *p* < 0.01 compared to the control group.

We determined at which stage of the viral life cycle VP acts upon via a time of addition assay. We treated SFTSV-infected Huh7 cells with VP at 2 h treatment windows, beginning with 2 h prior to the SFTSV infection, and up to 12 h post-infection. VP only significantly inhibited the SFTSV infection when added at the 0 h–2 h infection windows, suggesting the effect of VP is during the initial entry stage of the viral life cycle (Figure 2).

**Figure 3:**
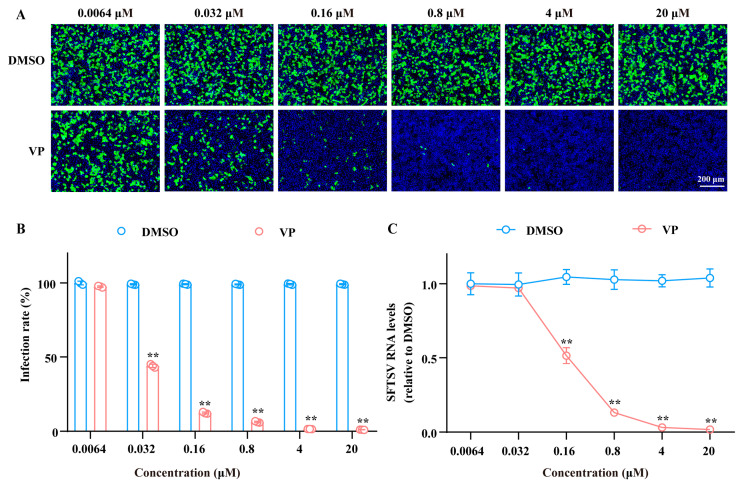
VP decreases SFTSV initial binding. Huh7 cells were co-incubated with SFTSV (MOI = 5) and the indicated concentrations (0.0064, 0.032, 0.16, 0.8, 4, and 20 μM) of VP or DMSO on ice for 1.5 h. A fraction of the infected and VP-treated cells were cultured at 37 °C for 24 h, with the SFTSV infection rate detected by IF. The SFTSV was stained with the primary anti-SFTSV NP mAb and goat anti-mouse Alexa Fluor™ 488 secondary antibody (green). Nuclei were stained with DAPI (blue) (**A**,**B**). The remaining cells were lysed and RT-qPCR was used to determine the amount of the bound virus (**C**). Data were shown as means with a SD of three independent experiments. Scale bar, 200 μm. ** *p* < 0.01 compared to the control group.

The SFTSV entry can be divided into the binding, endocytosis, and membrane fusion processes [24]. In order to reveal the mechanism of VP against SFTSV, we first investigated the effect of VP on the viral binding process. Huh7 cells were co-incubated with SFTSV and indicated concentrations of VP on ice for 1.5 h. The results demonstrated that VP impeded SFTSV binding to the cell surface in a dose-dependent manner both in viral protein expression and RNA synthesis, which is consistent with the antiviral effect observed in viral infection (Figure 3).

**Figure 4:**
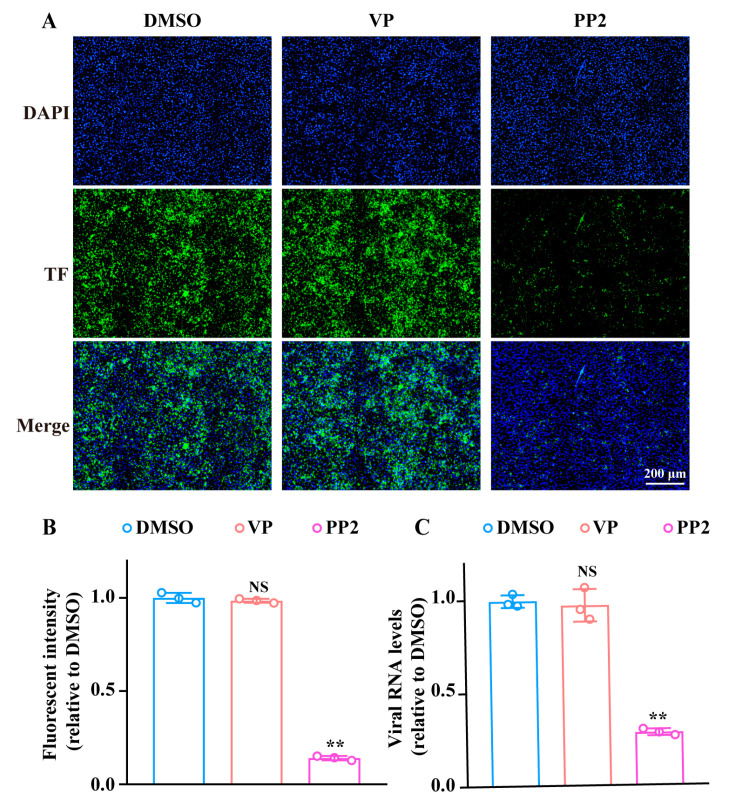
VP does not affect SFTSV endocytosis. (**A**,**B**) Huh7 cells were incubated with Alexa Fluor™ 488-labelled TF in the presence of VP (4 μM), PP2 (20 μM), or DMSO for 4 h. The fluorescent intensity of TF (green) was then examined by fluorescence microscopy. Nuclei were stained with DAPI (blue). (**C**) Huh7 cells were incubated with SFTSV on ice for 1.5 h. Then, the cellular supernatant was replaced with a fresh culture medium with VP (4 μM), PP2 (20 μM), or DMSO and the cells were incubated at 37 °C for 1 h. The intracellular SFTSV RNA levels were analyzed via RT-qPCR after being treated by proteinase K to remove the uninternalized SFTSV. Scale bar, 200 μm. ** *p* < 0.01 compared to the DMSO group. NS, no significance compared to the DMSO group.

In order to further clarify the effecting period, we detected the influence of VP on SFTSV endocytosis and membrane fusion. It is well acknowledged that SFTSV enters cells through the clathrin-mediated endocytosis pathway [25]. Therefore, we utilized the fluorescence-labeled transferrin (TF), a classic molecule internalized via the clathrin-mediated pathway. As shown in Figure 4A,B, no major difference of TF fluorescent intensity was observed between the VP-treated group and control, while PP2, a specific inhibitor of clathrin-mediated endocytosis, significantly decreased the uptake of TF, indicating VP did not affect SFTSV endocytosis. Moreover, we also evaluated the effect of VP on SFTSV endocytosis through RT-qPCR. Huh7 cells were incubated with the virus on ice for 1.5 h, and then the supernatant was replaced with fresh medium containing VP, and cells were maintained at 37 °C for 1 h, followed by the treatment of proteinase K to remove the uninternalized virus, and the intracellular SFTSV was determined. We observed the same lack of SFTSV RNA decrease via RT-PCR with VP treatment in this assay (Figure 4C) that we did with fluorescence intensity (Figure 4B).

**Figure 5:**
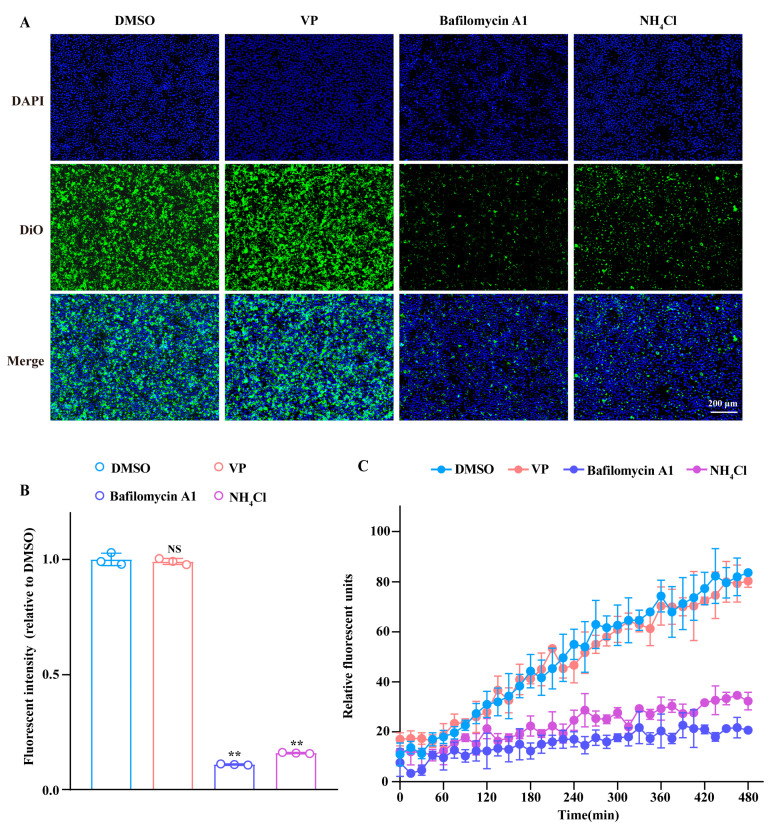
VP does not affect SFTSV membrane fusion. (**A**,**B**) Huh7 cells were inoculated with DiO-labelled SFTSV (SFTSV^DiO^, MOI = 1) in the presence of VP (4 μM), bafilomycin A1 (20 nM), NH_4_Cl (20 mM), or DMSO at 37 °C for 2 h. Then, the fluorescent intensity of DiO (green) was detected by the BioTek Lionheart FX Imaging Reader. Nuclei were stained with DAPI (blue). (**C**) Huh7 cells were incubated with SFTSV^DiO^ (MOI = 1) in the presence of VP (4 μM), bafilomycin A1 (20 nM), NH_4_Cl (20 mM), or DMSO. Then, the fluorescent units were collected every 15 min for a total of 33 cycles. Data were shown as means with a SD of three independent experiments. Scale bar, 200 μm. ** *p* < 0.01 compared to the DMSO group. NS, no significance compared to the DMSO group.

Subsequently, we used the DiO-labelled SFTSV (SFTSV^DiO^) to detect whether VP affected the SFTSV membrane fusion process, which was conducted according to our previous study [26]. As shown in Figure 5, in contrast with the significant reduction in DiO fluorescent intensity at 2 h post virus inoculation following bafilomycin A1 and NH_4_Cl treatment, no significant difference was observed between the VP-treated group and control. Moreover, the dynamic detection of the DiO fluorescence change further confirmed the fact that VP did not impede the SFTSV membrane fusion process.

### 3.3. VP Inhibits the SFTSV Binding Process by Inducing Viral Gn Protein Degradation

**Figure 6:**
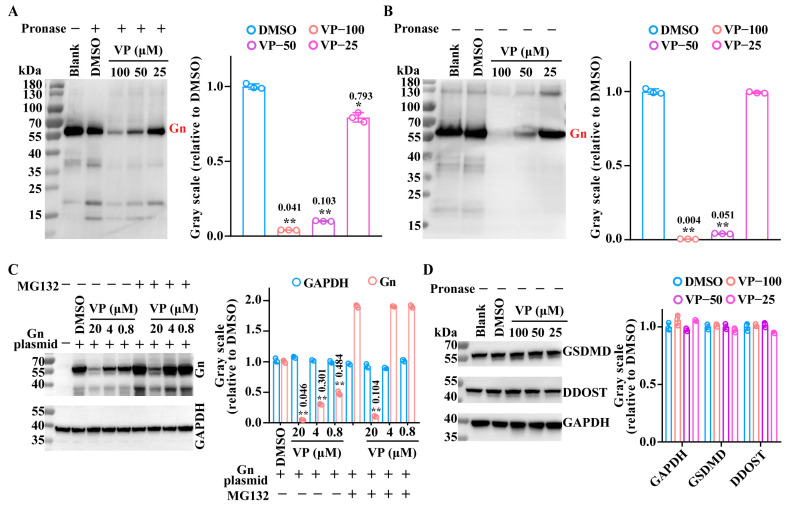
Degradation of the viral Gn protein by VP. (**A**) The Gn protein was pre-incubated with VP at the indicated concentrations (100 μM, 50 μM, and 25 μM) or DMSO for 1 h and subsequently digested using pronase for 30 min at room temperature. The influence of VP on Gn was analyzed by a Western blotting assay. (**B**) The Gn protein was incubated with VP at the indicated concentrations (100 μM, 50 μM, and 25 μM) or DMSO for 1.5 h, and the Gn was detected by a Western blotting assay. (**C**) The HeLa cells transfected with the Gn plasmid were treated with VP (20 μM, 4 μM, and 0.8 μM) and the proteasome inhibitor MG132 (10 μM) for 2 h. Then, the degradation effect was detected by a Western blotting assay. (**D**) The DDOST and GSDMD in cell lysates were incubated with the indicated concentrations of VP (100 μM, 50 μM, and 25 μM) for 1.5 h, and the influence of VP on DDOST and GSDMD was analyzed by a Western blotting assay. Data were shown as means with a SD of three independent experiments. ** *p* < 0.01 compared to the DMSO group. * *p* < 0.05 compared to the DMSO group.

It has been confirmed that the Gn protein plays an important role in the mediation of SFTSV binding. Thus, we used a drug affinity responsive target stability (DARTS) assay to detect the interaction between VP and the Gn protein. The DARTS assay represents a relatively rapid and direct method of verifying the interaction between molecule compounds and target proteins. Through interaction, the compound enhances the stability of the target protein and reduces its susceptibility to pronase hydrolysis, thereby protecting its structural integrity and functional properties [27]. Interestingly, the results of the DARTS assay indicated that the Gn protein was more degraded by pronase after the VP treatment (Figure 6A). Based on the results, we speculated that VP may induce the degradation of the viral Gn protein. Therefore, the SFTSV Gn protein was incubated only with VP individually and detected using a WB assay. In accordance with the results of the DARTS experiment, the incubation of the Gn protein with VP in the absence of pronase still resulted in the degradation of the Gn protein (Figure 6B). Furthermore, the VP-induced degradation of the Gn protein was also verified in HeLa cells that had been transfected with the Gn-expressing plasmid. The results demonstrated that VP could also induce the degradation of the Gn protein in HeLa cells. In addition, the proteasome inhibitor MG132 was unable to block this degradation. In order to ascertain the specificity of VP on Gn degradation, we also detected whether the compound could degrade cellular proteins such as dolichyl-diphosphooligosaccharide-protein glycosyltransferase non-catalytic subunit (DDOST), and gasdermin D (GSDMD). As shown in Figure 6D, VP was incapable of inducing the degradation of any of these three proteins.

### 3.4. VP Exerts Broad Spectrum Antiviral Activity Against a Wide Range of Viruses

**Figure 7:**
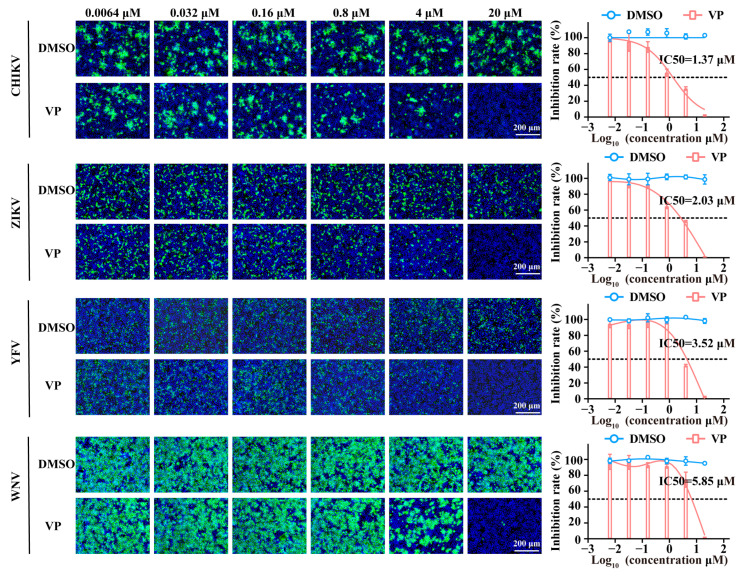
The broad-spectrum antiviral activity of VP. Huh7 cells were infected with CHIKV, ZIKV, YFV, and WNV at an MOI of 1 in the presence of VP or DMSO at the indicated concentrations. Then, the inhibitory effect was quantified by an IF assay. The SFTSV was stained with the primary anti-SFTSV NP mAb and goat anti-mouse Alexa Fluor™ 488 secondary antibody (green). Nuclei were stained with DAPI (blue). Data were shown as means with a SD of three independent experiments. Scale bar, 200 μm.

Finally, we evaluated the inhibitory effect of VP against other arboviruses. As shown in the results, the infection of the chikungunya virus (CHIKV), Zika virus (ZIKV), yellow fever virus (YFV), and West Nile virus (WNV) was markedly suppressed following VP treatment in a dose-dependent manner in Huh7 cells. The inhibition rate order of VP according to the IC_50_ values for these viruses is as follows: CHIKV (IC_50_: 1.37 μM), ZIKV (IC_50_: 2.03 μM), YFV (IC_50_: 3.52 μM), and WNV (IC_50_: 5.85 μM) (Figure 7).

## 4. Discussion

In recent years, there has been an escalating prevalence of SFTSV infection, accompanied by a rising fatality rate among SFTS patients, posing a serious public health concern worldwide. Consequently, it is urgent to develop specific and effective anti-SFTSV drugs to combat this emerging infectious disease. Among the compounds reported to possess anti-SFTSV activity, the broad-spectrum antiviral drugs, ribavirin and favipiravir, have been expected to have an ideal therapeutic effect on SFTS patients [28,29]. However, neither of the compounds achieved satisfactory results in clinical trials due to a limited efficacy and side effects [24,29]. Therefore, there are still no effective drugs available for the treatment of SFTSV infection.

The development of a high-throughput screening of FDA-approved marketing drugs has greatly accelerated the process of exploring and characterizing antiviral drugs against a wide range of viruses, such as the Lassa virus, Zika virus, and Ebola virus [30,31,32]. In the present study, we have observed, for the first time, that verteporfin (VP) exhibited a significant inhibition effect against SFTSV infection. As the second-generation porphyrin-based photosensitizer, VP has been approved by the FDA for the treatment of age-related macular degeneration in the clinics [17], thereby substantiating its safety and feasibility to be utilized in the human body as a potential antiviral agent.

Furthermore, using research platforms targeting different stages of the viral life cycle such as viral entry, replication, assembly, and release, we found that VP inhibited SFTSV infection at the entry stage by interfering with the binding to the cell surface. Binding is the initial stage of a viral infection, and the inhibition of viral binding serves to impede the establishment of infection at its very beginning stage, thus providing the compound the unique preventive advantage except for its therapeutic effect. Therefore, binding is a promising target in the development of antiviral drugs.

It has been demonstrated that the Gn protein plays key roles in SFTSV infection [33]. As an envelope glycoprotein, the Gn protein has been revealed to mediate the SFTSV binding process by interacting with cell-surface non-muscle myosin heavy chain IIA as well as dendritic cell-specific intercellular adhesion molecule-3-grabbing nonintegrin (DC-SIGN) [15,34]. Moreover, the Gn protein could promote SFTSV immune escape and infection by suppressing innate immunity [16]. Since VP impeded the binding process of SFTSV, we first examined the interaction between VP and Gn protein using the DARTS assay. Contrary to expectation, VP did not appear to offer any protection against pronase hydrolysis in the DARTS assay, indicating that no interaction between VP and the SFTSV Gn protein existed. However, we found surprisingly that VP was capable of inducing Gn protein degradation in the absence of pronase.

The conventional approach to drug development is predicated upon the identification of small molecules that are capable of binding to target proteins, thereby impeding their function [35,36]. Yet only a few classes of proteins can be modulated upon small molecule binding [37]. In recent years, a plethora of novel targeted protein degradation technologies, including PROteolysis TArgeting Chimeras (PROTACs), autophagy-targeting chimeras (AUTACs), autophagy-tethering compounds (ATTECs), lysosome targeting chimeras (LYTACs), and molecular degraders of extracellular proteins through the asialoglycoprotein receptor ASGPR (MoDE-As) have been developed to address these challenges [38]. However, to date, there have been no reports of small molecule compounds being capable of directly inducing protein degradation. Thus, the direct induction of Gn protein degradation by VP may represent a novel technique for drug discovery. Yet, the precise mechanisms involved in this phenomenon still need to be further elucidated and explored, and the in vivo antiviral activity at least in infection animal models are still required in subsequent studies.

The broad-spectrum antiviral activity of VP against CHIKV, ZIKV, YFV, and WNV makes us hypothesize that VP might induce the degradation of the functional viral E protein (flavivirus) or E2 protein (alphavirus) similar to the Gn of SFTSV, which plays important roles in the viral binding process. However, it should be noted that the VP-induced degradation of the SFTSV Gn protein in the DARTS assay could also potentially be attributed to VP blocking the binding of the antibody to the Gn protein. Moreover, it has been confirmed that glycosaminoglycans and lectins fulfil an important function in the binding process of SFTSV, CHIKV, ZIKV, YFV, and WNV [15,39,40]. Therefore, the universal antiviral effect may also be due to the VP blockage of the interaction between Gn, E, or E2 proteins with glycosaminoglycans or lectins. Nevertheless, further experiments need to be carried out to clarify the potential mechanism in future study.

In summary, the present study has identified VP to be a promising candidate compound for the treatment of SFTS patients. The compound suppressed SFTSV infection significantly by interfering with the viral binding process via directly inducing the degradation of the Gn protein. Moreover, we demonstrated the broad-spectrum antiviral activity of VP against WNV, ZIKV, YFV, and CHIKV. The findings provide novel insights for the development of anti-SFTSV drugs, especially for the preventive and therapeutic strategies for SFTS patients following the further detailed investigation and exploration.

## Figures and Tables

**Table 1:** Primer sequences.

Primer	Sequences
GAPDH	Forward primer: TGGGCTACACTGAGCACCAG
	Reverse primer: AAGTGGTCGTTGAGGGCAAT
SFTSV	Forward primer: TGGAGGCCTACTCTCTGTGG
	Reverse primer: AGCCACTTCACCCGAACATC

## Data Availability

Data are contained within the article.
